# Everybody's Page

**Published:** 1892-02-13

**Authors:** 


### EVERYBODY'S PAGE.

DR. J. N. BRAINERD, of Alma, Michigan, places a high therapeutic value on eucalyptol in some bronchial and pulmonary affections. His experience, derived from its use during a period of ten years, has led him to believe it to be of utility, though to a smaller degree, even in cases of tuberculosis.

---

THE consumption of alcohol in Germany is stated to have assumed such proportions as to alarm the authorities and to induce them to endeavour to regulate its sale by legislation. The encouragement of beer drinking by reducing the price of beer has been proposed as one remedial measure! We were not aware the German required any encouragement in this direction.

---

THE *Newspaper Review* is a distinct improvement upon many papers of its class. It gives interesting summaries from the various publications of the day, is well-arranged, well-printed, and entertaining besides. Seeing that it has to deal with newspapers of all shades of opinion, however, it is a pity to limit its circulation by showing so marked a political bias. It fulfils a useful purpose, and will in any care, we have no doubt, be widely read.

---

FROM the remotest ages human credulity has attributed occult qualities to various stones. The ill-luck supposed to attend the wearers of opals is proverbial. On the other hand there are many people whose friends and acquaintances would, perhaps, receive them with greater warmth if they were to wear emeralds, for this stone is believed by some to have the estimable qualities of making people agreeable and discreet! The origin of the various superstitions as to precious stones is probably traceable to Asia.

---

DR. DESROCHES has recently drawn attention in Canada in a forcible manner to the necessity of paying attention to the hygienic surroundings of children during their education. If his remarks are not original, they are at least practical and sensible. It cannot be said in this country that in the ardour after the inculcation of knowledge, the physical growth of our youth, whether boys or girls, is neglected. But it appears to have been necessary for Dr. Desroches to impress upon his fellow inhabitants in Montreal the fact that a young man of twenty years of age, however well equipped his intellect, may be of little use, either to the domestic circle or his country, if his physical powers are enfeebled by neglect.

THE successful cultivation of the camphor tree in Florida, where it is said to flourish so vigorously and grow so rapidly that there is a probability that in a few years' time there will be more camphor than orange trees in the country, should tend to bring down the high prices which have prevailed during the past few years.

---

ACCORDING to a French lady, woman is the most indefinable creature in the world. Amongst all the nations of the world you will not find any two who hold the same idea upon her. In Africa she is looked upon as a slave; in India and China as a machine; in Turkey she is viewed in the light of a pretty toy which must be kept under lock and key; in Spain as a dangerous enemy; in Russia as a being whose happiness is increased by a beating from time to time; in England as an equal to be respected, admired, and loved; and in France as a divinity to be adored.

---

THE statistics of the amount of sunshine, or rather the absence of sunshine during the past ten years, which have been published, are somewhat more mournful reading than statistics usually are. Londoners have come to be astonished, not at the absence of sunshine, but at the sight of the sun. That the inhabitants of this overgrown excrescence go about their duties with as light a heart as they do, when the sun is sometimes not to be seen for eight weeks on end, goes to show that the Englishman is not naturally morose. It is to his credit that he manages to be cheerful in spite of the ever-present gloom.

---

AFTER being told for many years that for the sick and those who cannot take solid food nothing is more nutritious and strength-sustaining than the various beef extracts which have from time to time been put on the market, we are assured in some quarters that these liquids are practically worthless as nutritives. The confiding patient has certainly hoped, if he has not quite believed, that these expensive luxuries must, owing to their costs be beneficial. Beneficial they probably have been to the manufacturers, and we will hope in some cases to invalids. Commercial enterprise has generally been equal to the demands upon it, and dessicated food is now held by some physicians to be of more nutritive value than the liquid foods which have so long held the field; there is still plenty of room for progress in the scientific manufacture of artificial foods.

